# Genetic Factors Associated with Intraocular Inflammation After Brolucizumab Administration in Patients with Exudative Age-Related Macular Degeneration

**DOI:** 10.3390/genes16070797

**Published:** 2025-07-01

**Authors:** Seigo Yoneyama, Yoichi Sakurada, Taiyo Shijo, Yoshiko Fukuda, Yumi Kotoda, Wataru Kikushima, Fumihiko Mabuchi, Kenji Kashiwagi

**Affiliations:** Department of Ophthalmology, Faculty of Medicine, University of Yamanashi, Yamanashi 409-3898, Japan; syoneyema@yamanashi.ac.jp (S.Y.); tshijoh@tdc.ac.jp (T.S.); ysugiyama@yamanashi.ac.jp (Y.F.); ykanai@yamanashi.ac.jp (Y.K.); wkikushima@yamanashi.ac.jp (W.K.); fmabuchi@icluoud.com (F.M.); kenjik@yamanashi.ac.jp (K.K.)

**Keywords:** age-related macular degeneration, intraocular inflammation, brolucizumab, CETP

## Abstract

**Purpose:** We aimed to investigate whether genetic variants susceptible to age-related macular degeneration (AMD) are associated with intraocular inflammation after brolucizumab administration in eyes that have exudative AMD. **Methods:** A total of 206 eyes from 206 patients (156 men/50 women, 74.0 ± 8.4 years; treatment-naïve, 128 [62.1%]; switching, 78 [37.9%]) were included in this study. All patients were treated with intravitreal brolucizumab at least once. The genotyping of *ARMS2 A69S* (rs10490924), *CFH I62V* (rs800292), *CFH* (rs1329428), *SKIV2L* (rs429608), *C3* (rs2241394), cholesteryl ester transfer protein (*CETP*) (rs3764261), and *ADAMTS9* (rs6795375) was performed using TaqMan technology. **Results:** Out of the 206 patients who were included, 21 eyes from 21 patients (10.2%) exhibited intraocular inflammation (IOI). Four (19.0%) exhibited severe IOI, including retinal vasculitis and/or retinal vascular occlusion, and 17 (81.0%) showed mild IOI. The frequency of the T allele of the *CETP* gene was significantly lower in patients who developed IOI compared to patients who did not develop IOI (T allele frequency: 9.5% vs. 23.5%, *p* = 0.036). After adjusting for confounding factors, the T allele remained significantly associated with protection against IOI (*p* = 0.028, 95% confidence interval: 0.098–0.88). **Conclusions:** The T allele of the *CETP* gene, a risk allele for AMD and the protective allele for atherosclerosis, may be associated with protection against IOI after brolucizumab administration in eyes that have exudative AMD.

## 1. Introduction

Age-related macular degeneration (AMD) is the leading cause of visual impairment and blindness in developed countries. It is estimated that 288 million people will be affected by 2040 [1]. Generally, the AMD stage is classified based on drusen size as an early, intermediate, or advanced stage [2]. Early AMD is characterized by small drusen less than 126 μm, and its risk of progression to advanced AMD is low. Intermediate AMD is characterized by drusen larger than 125 μm and/or retinal pigment epithelium (RPE) abnormalities. The risk of progression to advanced AMD is greater than early AMD or no AMD. Drusenoid pigment epithelial detachment (PED) is considered to be categorized as intermediate AMD. The risk of progression to advanced AMD is 30–50% in eyes with drusenoid PED [3]. There are two types of advanced AMD: dry and wet/exudative. The advanced form of dry AMD is called geographic atrophy and is characterized by complete atrophy of the outer retina and retinal pigment epithelium. The wet form is characterized by macular neovascularization originating from the choroid or retina. Type 1 and type 2 MNV originate from sub-RPE, and type 1 MNV is located in the sub-RPE space. Type 2 MNV breaks through RPE and is located above RPE. Type 3 MNV originates from the inner retina, progresses downward, and breaks through the RPE [4]. Apart from drusen-driven AMD, pachychoroid-driven AMD is characterized by pachychoroid features with macular neovascularization (MNV). Pachychoroid features are characterized by a focal or diffuse increase in choroidal thickness that is accounted for by the dilated choroidal vessels of the Haller layer, the attenuation of the inner choroid, and choroidal vascular hyperpermeability on indocyanine green angiography. The pachychoroid-driven MNV is called pachychoroid neovasculopathy.

The first line of treatment approved for neovascular AMD by the Food and Drug Administration (FDA) was photodynamic therapy using verteporfin. A large-scale randomized clinical trial, namely TAP (the treatment of age-related macular degeneration with photodynamic therapy), was reported in 1999 [5]. In this trial, 609 patients were randomly assigned to the PDT treatment group or the placebo group (no treatment group) and followed up over 24 months. At the 24-month visit, the best-corrected visual acuity (BCVA) had deteriorated by 19.6 letters and 13.4 letters from baseline in the placebo group and the PDT treatment group, suggesting that PDT was effective in slowing BCVA’s decline, but it could not improve BCVA in patients with neovascular AMD at any point. However, PDT is effective for the resolution of subretinal fluid in eyes with pachychoroid neovasculopathy and the occlusion of polypoidal lesions in eyes with PCV. The occlusion rate of polypoidal lesions was higher in the combination therapy involving the PDT and VEGF inhibitor than in the first-generation VEGF inhibitor, including ranibizumab and aflibercept. Moreover, PDT effectively decreases choroidal thickness in eyes with pachychoroid neovasculopathy. Therefore, it is still used in clinical practice, especially in Asian countries.

In 2004, the VISION study, a randomized clinical trial to evaluate pegaptanib in eyes with neovascular AMD using 1186 cohorts, was reported [6]. Pegaptanib was the first approved vascular endothelial growth factor (VEGF) inhibitor for the treatment of neovascular AMD by the FDA. There are five isoforms in VEGF-A. Pegaptanib was designed to selectively inhibit VEGF-A 165. In this study, the patients were assigned to the sham injection group and the pegaptanib treatment group. In addition, the pegaptanib treatment group was subdivided into three groups: 0.3 mg, 1.0 mg, and 3.0 mg. All patients were followed up every 6 weeks up to 54 weeks. During the 54-week visit, BCVA had declined by almost three lines from baseline in the placebo group. In the pegaptanib treatment group, BCVA declined by approximately 1–2 lines from baseline, irrespective of the dosing concentration, suggesting that it is insufficient to inhibit only VEGF-A 165 in eyes with neovascular AMD.

The advent of ranibizumab has revolutionized exudative AMD treatment. Ranibizumab, a recombinant, humanized, monoclonal antibody–antigen-binding fragment, was designed to inhibit all biologically active isoforms of VEGF-A, with a molecular weight of 48 kDa. Until the advent of brolucizumab, it had the smallest molecular weight of VEGF inhibitors commercially available. ANCHOR and MARINA, randomized clinical trials evaluating the efficacy of ranibizumab compared to a sham injection or PDT, showed a BCVA improvement of 6.6–11.3 letters in eyes with exudative AMD treated with monthly ranibizumab administration over 24 months, irrespective of the AMD subtype [7,8]. Subsequently, the VIEW study demonstrated non-inferior visual gains after every 8 weeks of administering aflibercept 2 mg, following three consecutive monthly doses, compared with monthly ranibizumab dosing [9]. However, in clinical practice, it is a great burden for patients and physicians to administer the fixed monthly or bimonthly administration of VEGF inhibitors. In 2009, the PRONTO study investigated whether variable ranibizumab dosing regimens could be an effective treatment regimen for improving BCVA in eyes with neovascular AMD [10]. Patients were followed up monthly after three consecutive monthly doses of ranibizumab. If the retreatment criteria were fulfilled on each visit, an additional injection of ranibizumab was performed. At the 24-month visit, the mean BCVA improved by 11.1 letters with a total of 10 injections, including 3 loading injections. This was the first study to demonstrate the efficacy of the variable dosing administration of VEGF inhibitors for neovascular AMD, and this regimen is called the “as-needed” or “pro re nata” regimen. The Seven-Up study is an extension study of ANCHOR/MARINA and the Open-Label Extension Trial of Ranibizumab for Choroidal Neovascularization Secondary to Age-Related Macular Degeneration (HORIZON) [11]. In this study, patients were followed up with as-needed additional injections at the physicians’ discretion. During the mean follow-up of 7.3 years, one-third of patients demonstrated good visual outcomes, and another one-third of patients declined by 15 letters or more. At the 60-month visit, the mean BCVA returned to the baseline levels with a mean of four additional injections per year, suggesting that strict management is required in neovascular AMD treatment under an as-needed regimen because the regimen is likely to experience undertreatment. The treat-and-extend regimen is a proactive remedy to administer VEGF inhibitors in the loading phase, and thereafter, the interval of VEGF inhibitor administration can be changed with the ability to monitor the presence or absence of recurrent exudation in the OCT. The prospective trial, TREX-AMD, comparing treat-and-extend vs. monthly dosing in one-year outcomes of neovascular AMD using ranibizumab, included 60 patients (with a mean age of 77 years (range 59–96), female/male: 38/22, mean BCVA 20/60) [12]. The patients were randomly assigned to a monthly dosing group (20 patients) and a treat-and-extend group (40 patients). The treat-and-extend protocol uses monthly injections until signs of exudation have been resolved. Then, the interval between treatments is lengthened, typically sequentially by 1- to 2-week intervals as long as there are no signs of recurrent exudation. When recurrent disease is detected, the treatment interval is shortened. The administration of ranibizumab is conducted at every visit, and the time between visits is individualized. In this trial, the treat-and-extend group showed significantly improved BCVA with a similar visual gain to the monthly dosing group (treat-and-extend group: 10.5 letters vs. monthly dosing group: 9.3 letters), and the mean number of injections during the 12-month follow-up period was 10.1, which is significantly fewer compared with the monthly dosing method. To date, the treat-and-extend regimen has been the most used treatment regimen for neovascular AMD in the world; however, this regimen might be a form of over-treatment for some patients.

Although various revisions have been conducted in the treatment regimen of neovascular AMD, VEGF inhibitors are currently expected to have a longer duration of action to reduce the potential treatment burden on patients and physicians.

Brolucizumab, an approximately 26 kDa single-chain antibody fragment, has the smallest molecular weight among commercially available VEGF inhibitors and achieves stability and solubility at high doses in a single 50 µL intravitreal injection. In a single 0.05 mL administration to the vitreous cavity, the equivalent molecular dose of brolucizumab was approximately 10 times greater than that of aflibercept and approximately 20 times greater than that of ranibizumab [13]. In the HAWK and HARRIER trials, brolucizumab at 6 mg demonstrated non-inferior visual improvements compared with aflibercept (2 mg), and 65% of eyes treated with 6 mg of brolucizumab achieved a prolonged treatment interval of 12 weeks. Although previous pivotal clinical trials reported local adverse events, including intraocular inflammation (IOI) in the form of non-infectious ocular inflammation or uveitis, its incidence was low, so it was not focused on. However, IOI becomes a major concern when associated with brolucizumab administration. In the HAWK and HARRIER studies, the incidence of IOI was higher with 6 mg (4%) brolucizumab than with 2 mg (1%) aflibercept [14]. However, several real-world studies have reported high incidences of IOI. Several studies have investigated the risk factors for IOI after brolucizumab administration, including systemic diseases and cytokine levels in the anterior chamber [15,16,17]. However, no studies have investigated the genetic factors associated with IOI after brolucizumab administration. In the present study, we genotyped seven major genetic variants associated with neovascular AMD and examined the association between these variants and IOI after brolucizumab administration.

## 2. Methods

This was a single-center, retrospective, observational study. The medical records of patients with AMD/PCV who were treated with brolucizumab between August 2020 and July 2024 were retrospectively reviewed. This study was approved by the Institutional Review Board of the University of Yamanashi and was conducted in accordance with the Declaration of Helsinki (approval number: 2205). Written informed consent was obtained from all participants before treatment initiation.

## 3. Participants

All patients treated with brolucizumab and followed up for at least 6 months were enrolled in this study.

At the initial visit, all patients underwent a comprehensive examination, including the measurement of best-corrected visual acuity using a Landolt chart, slit-lamp biomicroscopy with or without a 78-diopter lens, intraocular pressure measurements, fundus photography, swept-source and spectral-domain optical coherence tomography (OCT), and fluorescein/indocyanine green angiography (FA/ICGA). The diagnosis of neovascular AMD or PCV was made using multimodal imaging, including FA/ICGA and SS/SD-OCT. PCV is defined as the presence of hot spot(s) on ICGA corresponding to the retinal pigment epithelial protrusion or dome-shaped PED, irrespective of the presence or absence of a branching vascular network. Neovascular AMD was defined as occult or classic leakage on FA without hot spots corresponding to polypoidal lesion(s) on ICGA.

Treatment-naïve patients with AMD/PCV were administered three consecutive monthly injections of brolucizumab (6.0 mg/0.05 mL) and were followed up monthly for up to 12 months, as previously reported [18]. In patients who switched to brolucizumab, a treat-and-extend regimen was administered without a loading regimen. In this switching regimen, the treatment interval was determined based on the interval of prior VEGF inhibitor treatment, as previously described [19]. The VEGF inhibitor used before switching to brolucizumab was aflibercept 2 mg in all switching patients. Patients with a history of retinal vascular occlusion or uveitis were excluded. Eyes with other retinal pathologies, including retinal angiomatous proliferation, macular neovascularization secondary to high myopia, angioid streaks, or inflammatory diseases, including multifocal choroiditis or punctate inner choroidopathy, were also excluded because these disorders were treated with 2 mg of ranibizumab or aflibercept in our hospital.

## 4. Definition of IOI

IOI was defined as any type of inflammation of the anterior chamber and/or vitritis, retinal vasculitis, or retinal vascular occlusion, except for infectious endophthalmitis. IOI was classified into two types: mild and severe.

The mild type was defined as inflammation without retinal vasculitis or retinal vascular occlusion, including anterior chamber inflammation and vitritis. The severe type was defined as retinal inflammation, including retinal vasculitis or retinal vascular occlusion [20]. Ultra-wide fluorescein angiography and color fundus photography confirmed the presence or absence of retinal vasculitis or vascular occlusions. SS-OCT is also used to detect the ischemic inner retina, which usually shows a hyperreflective inner retina.

## 5. Statistical Analysis

Statistical analysis was performed using SPSS 13 software. Chi-square and Mann–Whitney U tests were used to test for differences in categorical and continuous variables between the two groups. Multiple regression analysis was used to determine the factors associated with IOI. Statistical significance was defined as *p* < 0.05.

## 6. Results

This study included 206 patients (men/women: 156 (75.6%)/50 (24.4%); mean age, 74.0 ± 8.4 years). Table 1 shows the demographic and genetic characteristics of patients with and without IOI. The T allele of the cholesteryl ester transfer protein (*CETP*) rs3764261 was significantly protective against IOI development (*p* = 0.038, chi-square test), although the incidence of IOI did not reach statistical significance (*p* = 0.055) between the switching group and the treatment-naïve group. Table 2 shows the results of multivariate regression analysis associated with IOI. The T allele of *CETP* rs3764261 was significantly associated with a decreased risk of IOI (*p* = 0.028; hazard ratio, 0.29; 95% confidence interval: 0.098–0.88). Table 3 shows a comparison between severe and mild IOI. In both groups, the median number of injections was one when IOI first developed. The severe IOI group had a significantly high frequency of women and risk alleles of *CFH* rs1329428 and *C3* rs2241394. However, multivariate regression analysis showed that none of the variables, including female sex, CFH, and C3, were associated with severe IOI (all *p* > 0.05). This might be due to the small number of the severe IOI group.

## 7. Discussion

In 2022 and 2024, 8 mg of brolucizumab, faricimab, and aflibercept were commercially available for eyes with neovascular AMD and diabetic macular edema, respectively. They are called second-generation VEGF inhibitors because they can provide a higher dose in a single injection compared with 2 mg of aflibercept or ranibizumab. In large-scale studies, including TENAYA/LUCERN and PULSAR, it was not reported that IOI was a common topical adverse event, including vitritis (~1.0%) and uveitis (~1.0%). However, several studies in the real world have reported IOI after the administration of these VEGF inhibitors, including 8 mg of faricimab and aflibercept [21,22,23]. In the report regarding faricimab-associated IOI, IOI appeared as vitritis and/or iritis, and the prognosis of most eyes was favorable after topical steroid treatment. As a form of faricimab-associated IOI, 8 mg of aflibercept-associated IOI appears as mild vitritis the prognosis of which is favorable. Although some eyes exhibited severe IOI after brolucizumab administration, IOI may be a common adverse event after the administration of second-generation VEGF inhibitors. Although an excessive and rapid reduction in VEGF levels in vitreous humor is associated with IOI, the underlying mechanism is not fully understood.

Several studies have investigated the incidence and risk of IOI following brolucizumab administration in patients with exudative AMD. They reported that the incidence of IOI is approximately 10% in Asian populations, which is higher than that reported in Caucasians; female sex, a history of diabetes mellitus (DM), and older age were also associated with IOI [15,24,25]. However, there was no significant association between IOI and DM, female sex, and age in this study. In addition to demographic and clinical characteristics, we investigated whether seven major AMD-susceptible genetic variants are associated with IOI. The major genetic variants associated with the alternative complement pathway were not associated with IOI; however, the T allele of *CETP* rs3764261 on chromosome 16 was associated with protection against IOI. *CETP* is a hydrophobic glycoprotein secreted from liver Kupffer cells into plasma high-density lipoproteins (HDLs). It mediates the transfer of cholesteryl esters from HDL to very-low-density lipoproteins and low-density lipoproteins [26]. The T (risk) allele of rs3764261 in the *CETP* gene was reported to be associated with AMD in a genome-wide association study [27]. The T allele of rs3764261 is associated with high HDL cholesterol levels. By contrast, the C (protective) allele of rs3764261 has been associated with atherosclerosis and cardiovascular disease. VEGF is primarily secreted by the retinal pigment epithelium and plays a pivotal role in retinal homeostasis, including the formation and maintenance of retinal vessels [28]. Rapid and excessive reductions in VEGF might cause the inflammation of ocular tissues, including the uvea and retinal vessels, especially in eyes with retinal vascular atherosclerosis. Retinal vascular atherosclerosis might progress more in carriers of the C allele of rs3764261 than in carriers of the T allele, like systemic atherosclerosis. Therefore, IOI may be more likely to occur in carriers of the C allele than in those of the T allele.

In addition to the association between IOI and genetic factors, we investigated the differences between severe and mild IOI. Female sex and the risk alleles of *CFH* rs1329428 and *C3* rs2241394 (*p* = 0.02, 0.047, and 0.037, respectively) were associated with severe IOI. CFH and C3 were strongly associated with AMD through an alternative pathway of complement activation. However, multivariate regression analysis revealed that none of these variables were associated with severe IOI.

The present study has several limitations. First, it is a retrospective study; therefore, it includes both treatment-naïve patients and those with a history of other forms of VEGF inhibitor treatment. In this study, patients were included with at least a 6-month follow-up. The number of brolucizumab injections was not the same between treatment-naïve eyes and eyes with a history of prior treatment. Second, although the total number of patients was more than 200, the sample size for IOI, especially for severe IOI, was small because the proportions of IOI and severe IOI were approximately 10% and 2%, respectively. Large-scale randomized studies are needed to confirm or refute these preliminary findings. In addition, it is interesting to investigate whether variants of the CETP gene are also associated with IOI alongside other second-generation VEGF inhibitors. Including 8 mg faricimab and aflibercept.

In summary, the C allele of the CETP gene might be associated with IOI after brolucizumab administration for exudative AMD.

## Figures and Tables

**Table 1:** Comparison between patients with or without developing intraocular inflammation.

	IOI (+) (n = 21)	IOI (−) (n = 185)	*p*-Value
Age (years)	72.8 ± 6.5	74.1 ± 8.6	0.37
Gender (male)	15 (71.4%)	141 (76.2%)	0.63
AMD subtype (tAMD vs. PCV)	5:16	81:104	0.078
HT	9 (42.9%)	104 (56.2%)	0.24
DM	3 (14.3%)	29 (15.7%)	0.87
Smoking (never/past/current)	6:10:5	60:97:28	0.96
Switch	12 (57.1%)	66 (35.7%)	0.055
*ARMS2 A69S* (rs10490924) T allele frequency	57.1%	59.5%	0.77
*CFH I62V* (rs800292) G allele frequency	76.2%	74.3%	0.79
*CFH* (rs1329428) C allele frequency	73.8%	65.1%	0.26
*SKIV2L* (rs429608) A allele frequency	9.5%	9.5%	0.99
*C3* (rs2241394) G allele frequency	2.4%	6.2%	0.31
*CETP* (rs3764261) T allele frequency	9.5%	23.5%	0.038
*ADAMTS9* (rs6795735) C allele frequency	16.7%	13.5%	0.57
	β-coefficient	*p*-value	Hazard ratio (95% confidence interval)
*CETP* (T allele)	0.29	0.028	0.29 (0.098–0.88)
*ARMS2 A69S* (rs10490924) T allele frequency	57.1%	59.5%	0.77
*CFH I62V* (rs800292) G allele frequency	76.2%	74.3%	0.79
*CFH* (rs1329428) C allele frequency	73.8%	65.1%	0.26
*SKIV2L* (rs429608) A allele frequency	9.5%	9.5%	0.99
*C3* (rs2241394) G allele frequency	2.4%	6.2%	0.31
*CETP* (rs3764261) T allele frequency	9.5%	23.5%	0.038
*ADAMTS9* (rs6795735) C allele frequency	16.7%	13.5%	0.57

AMD: age-related macular degeneration; DM: diabetes mellitus; HT: hypertension; IOI: intraocular inflammation; PCV: polypoidal choroidal vasculopathy. HT/DM was defined according to the presence or absence of a history of treatment.

**Table 2:** Multivariate logistic regression analysis associated with intraocular inflammation.

	β-Coefficient	*p*-Value	Hazard Ratio (95% Confidence Interval)
*CETP* (T allele)	0.29	0.028	0.29 (0.098–0.88)

**Table 3:** Comparison between severe and mild IOI.

	Severe (n = 4)	Mild (n = 17)	*p*-Value
Age	76.3 ± 8.1	71.9 ± 6.1	0.47
Gender (male)	1 (25.0%)	14 (82.4%)	0.02
AMD subtype (tAMD vs. PCV)	1:3	4:13	0.29
HT	2 (50.0%)	7 (41.2%)	0.41
DM	0 (0%)	3 (15.7%)	0.36
Switch	2 (50.0%)	10 (58.8%)	0.75
*ARMS2 A69S* (rs10490924) T allele frequency	7:1 87.5%	17:17 50.0%	0.054
*CFH I62V* (rs800292) G allele frequency	8:0 100%	24:10 70.6%	0.079
*CFH* (rs1329428) C allele frequency	8:0 100%	22:12 67.6%	0.047
*SKIV2L* (rs429608) A allele frequency	0:8 0%	4:30 11.8%	0.31
*C3* (rs2241394) G allele frequency	1:7 12.5%	0:34 0%	0.037
*CETP* (rs3764261) T allele frequency	0:8 0%	4:30 11.8%	0.31
*ADAMTS9* (rs6795735) C allele frequency	1:7 12.5%	6:28 17.6%	0.73

## Data Availability

The data cannot be shared because they are included in the genetic information.

## References

[1] Wong W.L., Su X., Li X., Cheung C.M., Klein R., Cheng C.Y., Wong T.Y. (2014). Global prevalence of age-related macular degeneration and disease burden projection for 2020 and 2040: A systematic review and meta-analysis. Lancet Glob. Health.

[2] Sakurada Y., Tanaka K., Fragiotta S. (2023). Differentiating drusen and drusenoid deposits subtypes on multimodal imaging and risk of advanced age-related macular degeneration. Jpn. J. Ophthalmol..

[3] Shijo T., Sakurada Y., Tanaka K., Miki A., Sugiyama A., Onoe H., Chubachi A., Kikushima W., Wakatsuki Y., Yoneyama S. (2022). Incidence and risk of advanced age-related macular degeneration in eyes with drusenoid pigment epithelial detachment. Sci. Rep..

[4] Spaide R.F., Jaffe G.J., Sarraf D., Freund K.B., Sadda S.R., Staurenghi G., Waheed N.K., Chakravarthy U., Rosenfeld P.J., Holz F.G. (2020). Consensus Nomenclature for Reporting Neovascular Age-Related Macular Degeneration Data: Consensus on Neovascular Age-Related Macular Degeneration Nomenclature Study Group. Ophthalmology.

[5] Treatment of Age-Related Macular Degeneration with Photodynamic Therapy (TAP) Study Group (1999). Photodynamic therapy of subfoveal choroidal neovascularization in age-related macular degeneration with verteporfin: One-year results of 2 randomized clinical trials--TAP report. Arch. Ophthalmol..

[6] Gragoudas E.S., Adamis A.P., Cunningham E.T., Feinsod M., Guyer D.R., VEGF Inhibition Study in Ocular Neovascularization Clinical Trial Group (2004). Pegaptanib for neovascular age-related macular degeneration. N. Engl. J. Med..

[7] Brown D.M., Kaiser P.K., Michels M., Soubrane G., Heier J.S., Kim R.Y., Sy J.P., Schneider S., ANCHOR Study Group (2006). Ranibizumab versus verteporfin for neovascular age-related macular degeneration. N. Engl. J. Med..

[8] Rosenfeld P.J., Brown D.M., Heier J.S., Boyer D.S., Kaiser P.K., Chung C.Y., Kim R.Y., MARINA Study Group (2006). Ranibizumab for neovascular age-related macular degeneration. N. Engl. J. Med..

[9] Heier J.S., Brown D.M., Chong V., Korobelnik J.F., Kaiser P.K., Nguyen Q.D., Kirchhof B., Ho A., Ogura Y., Yancopoulos G.D. (2012). Intravitreal aflibercept (VEGF trap-eye) in wet age-related macular degeneration. Ophthalmology.

[10] Lalwani G.A., Rosenfeld P.J., Fung A.E., Dubovy S.R., Michels S., Feuer W., Davis J.L., Flynn H.W., Esquiabro M. (2009). A variable-dosing regimen with intravitreal ranibizumab for neovascular age-related macular degeneration: Year 2 of the PrONTO Study. Am. J. Ophthalmol..

[11] Rofagha S., Bhisitkul R.B., Boyer D.S., Sadda S.R., Zhang K., SEVEN-UP Study Group (2013). Seven-year outcomes in ranibizumab-treated patients in ANCHOR, MARINA, and HORIZON: A multicenter cohort study (SEVEN-UP). Ophthalmology.

[12] Wykoff C.C., Croft D.E., Brown D.M., Wang R., Payne J.F., Clark L., Abdelfattah N.S., Sadda S.R., TREX-AMD Study Group (2015). Prospective Trial of Treat-and-Extend versus Monthly Dosing for Neovascular Age-Related Macular Degeneration: TREX-AMD 1-Year Results. Ophthalmology.

[13] Nguyen Q.D., Das A., Do D.V., Dugel P.U., Gomes A., Holz F.G., Koh A., Pan C.K., Sepah Y.J., Patel N. (2020). Brolucizumab: Evolution through Preclinical and Clinical Studies and the Implications for the Management of Neovascular Age-Related Macular Degeneration. Ophthalmology.

[14] Dugel P.U., Singh R.P., Koh A., Ogura Y., Weissgerber G., Gedif K., Jaffe G.J., Tadayoni R., Schmidt-Erfurth U., Holz F.G. (2021). HAWK and HARRIER: Ninety-Six-Week Outcomes from the Phase 3 Trials of Brolucizumab for Neovascular Age-Related Macular Degeneration. Ophthalmology.

[15] Mukai R., Matsumoto H., Akiyama H. (2021). Risk factors for emerging intraocular inflammation after intravitreal brolucizumab injection for age-related macular degeneration. PLoS ONE.

[16] Hashimoto Y., Inoda S., Takahashi H., Takahashi R., Yoshida H., Fujino Y., Sakamoto S., Kawashima H., Yanagi Y. (2024). Factors Associated with Intraocular Inflammation in Neovascular Age-Related Macular Degeneration Patients Treated with Brolucizumab. Investig. Ophthalmol. Vis. Sci..

[17] Ruamviboonsuk V., Kongwattananon W., Chuaypen N. (2025). Changes in Aqueous Humor Cytokine Profile Following Intravitreal Brolucizumab Injection. Clin. Ophthalmol..

[18] Fukuda Y., Sakurada Y., Matsubara M., Kotoda Y., Kasai Y., Sugiyama A., Kashiwagi K. (2023). Comparison of one-year outcomes between as-needed brolucizumab and aflibercept for polypoidal choroidal vasculopathy. Jpn. J. Ophthalmol..

[19] Kikushima W., Sakurada Y., Fukuda Y., Matsubara M., Kotoda Y., Sugiyama A., Kashiwagi K. (2023). A Treat-and-Extend Regimen of Intravitreal Brolucizumab for Exudative Age-Related Macular Degeneration Refractory to Aflibercept: A 12-Month Result. Pharmaceuticals.

[20] Sadda S.R., Holz F.G., Staurenghi G., Invernizzi A., Arnold J., Tadayoni R. (2022). The Importance of Imaging to Identify Early Signs of Intraocular Inflammation Expert Opinion for Brolucizumab. Ophthalmologica.

[21] Hoffmann L., Michels S., Eandi C., Karam M.A., Figueiredo E.C.O., Hatz K. (2024). Aflibercept high-dose (8mg) related intraocular inflammation (IOI)—A case series. BMC Ophthalmol..

[22] Ben Ghezala I., Gabrielle P.H., Sibert M., Steinberg L.A., Dautriche A., Arnould L., Creuzot-Garcher C. (2025). Severe Intraocular Inflammation After Intravitreal Injection of Faricimab: A Single-Site Case Series of Six Patients. Am. J. Ophthalmol..

[23] Montesel A., Sen S., Preston E., Patel P.J., Huemer J., Hamilton R.D., Nicholson L., Papasavvas I., Tucker W.R., Yeung I. (2025). Intraocular Inflammation Associated with Faricimab Therapy: One-Year Real-World Outcomes. Retina.

[24] Inoda S., Takahashi H., Maruyama-Inoue M., Ikeda S., Sekiryu T., Itagaki K., Matsumoto H., Mukai R., Nagai Y., Ohnaka M. (2024). Incidence and Risk Factors of Intraocular Inflammation After Brolucizumab Treatment in Japan: A Multicenter Age-Related Macular Degeneration Study. Retina.

[25] Park H.S., Lee S.W., Park H., Lee N.K., Kim Y.J., Lee C.S., Byeon S.H., Kim S.S. (2024). Incidence of intraocular inflammation and its risk factors in patients treated with brolucizumab: A nationwide cohort study. Sci. Rep..

[26] Zhou H., Li Z., Silver D.L., Jiang X.C. (2006). Cholesteryl ester transfer protein (CETP) expression enhances HDL cholesteryl ester liver delivery, which is independent of scavenger receptor BI, LDL receptor related protein and possibly LDL receptor. Biochim. Biophys. Acta.

[27] Chen W., Stambolian D., Edwards A.O., Branham K.E., Othman M., Jakobsdottir J., Tosakulwong N., Pericak-Vance M.A., Campochiaro P.A., Klein M.L. (2010). Genetic variants near TIMP3 and high-density lipoprotein-associated loci influence susceptibility to age-related macular degeneration. Proc. Natl. Acad. Sci. USA.

[28] Torczynski E. (1982). Normal and abnormal ocular development in man. Prog. Clin. Biol. Res..

